# Survival Associations between Patient Age and Treatment Modality in Olfactory Neuroblastoma: A Retrospective Population-Based Study

**DOI:** 10.3390/jcm10122685

**Published:** 2021-06-18

**Authors:** Andre J. Burnham, Phillip A. Burnham, Edwin M. Horwitz

**Affiliations:** 1 Department of Pediatrics, Emory University School of Medicine, Atlanta, GA 30322, USA; andre.joseph.burnham@emory.edu; 2 Aflac Cancer & Blood Disorders Center, Children’s Healthcare of Atlanta, Atlanta, GA 30322, USA; 3 Vermont Complex Systems Center, University of Vermont, Burlington, VT 05405, USA; phillip.burnham@uvm.edu

**Keywords:** olfactory neuroblastoma, esthesioneuroblastoma, head and neck cancer, chemotherapy, pediatric cancer

## Abstract

Olfactory neuroblastoma (ONB) is a rare neuroepithelial-derived malignancy that usually presents in the nasal cavity. The rarity of ONB has led to conflicting reports regarding associations of patient age and ONB survival and outcome. Moreover, long-term outcomes of chemotherapy and other treatment modalities are speculated. Here, we aimed to compare survival outcomes across age groups through time and determine associations between treatment modality and survival. In this retrospective population-based study, we analyzed the SEER 2000–2016 Database for patients with ONB tumors. Using Kaplan–Meier survival analysis, a significant effect of age and cancer-specific survival (CSS) was observed; geriatric ONB patients had the lowest CSS overall. Generalized linear models and survival analyses demonstrated that CSS of the pediatric patient population was similar to the geriatric group through 100 months but plateaued thereafter and was the highest of all age groups. Radiation and surgery were associated with increased CSS, while chemotherapy was associated with decreased CSS. GLM results showed that tumor grade, stage and lymph node involvement had no CSS associations with age or treatment modality. Our results provide insight for future investigations of long-term outcomes associated with ONB patient age and treatment modality, and we conclude that survival statistics of ONB patients should be analyzed in terms of trends through time rather than fixed in time.

## 1. Introduction

First described by Berger et al. in 1924 [1], olfactory neuroblastoma (ONB), also known as esthesioneuroblastoma, is a rare form of cancer believed to arise from neuroepithelia cells of the cribriform plate, nasal septum, and the middle and superior turbinates [2,3,4], Incidence of this malignancy is estimated at approximately 0.4 in one million, and tumors are most common in those above the age of 60, although all ages of patients may be affected [4,5,6], ONB usually presents in the nasal cavity, but may extend to the sinuses, orbital region and the brain; in advanced cases, ONB may metastasize to regional lymph nodes and systemic organs (e.g., lung, liver, and brain) [7,8], ONB may result in severe morbidities and patient mortality; other symptoms include loss of smell or eyesight, shortness of breath, nose bleeding, and headaches [3,4].

Due to the very low incidence of ONB, there is much debate regarding the effects of patient age on ONB patient outcomes, especially in pediatric populations [6,9,10,11,12,13,14,15]. Some investigators have suggested that children have a higher risk of mortality [9,10,11,12,13], while other studies demonstrate that the survival of adult and pediatric ONB patients are largely similar [6,14,15].

In addition to long-term outcomes of ONB patient age groups, treatment modality and the use of chemotherapy (CT) is another area of ongoing research. The standard of care for ONB involves surgical resection of the tumor and radiation therapy (RT) [8], both of which have been shown to improve patient outcomes and long-term survival [6,16]. However, the efficacy of CT treatment on ONB patient outcomes is largely speculated [8]. Some retrospective studies have reported positive patient outcomes in response to CT [17,18], while others have observed that CT is associated with poor patient outcomes [19,20,21], In a recent epidemiological survey, CT treatment was associated with poor ONB patient outcomes and reduced survival [21]. However, the long-term effects associated with CT and other treatment modalities on patient age groups remain unknown. Understanding the risk factors and long-term outcomes associated with age at diagnosis and CT is important for both patients and providers. Thus, there is a need for continued investigation of ONB patient outcomes within various age demographics.

Herein, we used historical data to compare long-term survival outcomes in pediatric, middle-age, and geriatric ONB patient populations. To elucidate survival outcomes among pediatric patients, we evaluated changes in survival data both statically and through time in all age groups. We further investigated the impact of CT and other treatment modalities on cancer-specific survival (CSS) in patients of various age demographics. This study provides valuable clinical insight and surveys the largest population and geographical coverage of ONB patients within the Surveillance Epidemiology and End Results (SEER) Database.

## 2. Materials and Methods

The Surveillance Epidemiology and End Results (SEER) 2000–2016 Database was analyzed for ONB cases (classification 9522-3). We selected the SEER 2000–2016 database was analyzed, as this database presents the largest geographic coverage available (27.8% of the U.S. population) and represents a recent patient population within the SEER Database; in addition, chemotherapy data were no longer reported after 2016. Cases were included in our analysis if they were unique and had primary tumors in the nasal cavity, maxillary sinus, ethmoid sinus, frontal sinus, sphenoid sinus, overlapping lesion of accessory sinuses, and accessory sinus, and these inclusion criteria were derived from previous studies [6,16,21]. A total of 882 cases met these criteria. Cancer-specific survival (CSS) was the primary outcome measured; CSS is defined by SEER as deaths attributed to the cancer of interest and treated as events, while deaths from other causes are treated as censored observations. Multivariable Cox proportional hazards regression and univariable and multivariable generalized linear models (GLM) were used for additional analyses. We also report patient demographic data (age, sex, race), disease characteristics (grade, stage, lymph node involvement), and treatment modality (chemotherapy, radiation, surgery). Patient demographics and characteristics of the study population are provided.

The tumor grading system used in this study, as recorded in the SEER database, is reported on a scale from I to IV, with grade I designated as well-differentiated, grade II as moderately differentiated, grade III as poorly differentiated, and grade IV as undifferentiated. This grading scheme roughly corresponds to the Hyams grading scale. In addition, the SEER historical staging system was used, which refers to tumors that manifested as either localized, regional, or distant diseases.

All data cleaning and analysis were conducted using the R programming language (R Core Team, version 3.6.2). In order to examine the relative risk and probability of survival of individuals across age at diagnoses, sex, race, treatment modality (CT, RT, or surgery) and disease severity (tumor grade, stage, lymph node involvement and distant disease), we computed Cox proportional hazards regression model (survival package, v3.1-8) and Kaplan–Meier survivor curves (survival package, v3.1-8). Corresponding forest plots were drawn using the “forestmodel” package (v0.6.2). Log-rank tests were conducted to examine the statistical significance between the above-mentioned Kaplan–Meier curves using the “survdiff” function (survival package, v3.1-8). The alpha level for all statistical analyses = 0.05.

To examine the important interaction effects of several variables of interest on survival, we conducted a series of generalized linear models. With survival as a binary variable, we used binomial distributions with a link = “logit” function in the “lme4” package (v1.1-23). Significance for all GLMs was determined using a likelihood ratio test (car package, v3.0-6). To further examine the survival dynamics between the pediatric age group vs. all other age groups through time, we and conducted a GLM computing the effect of age group (0–20 vs. 21+), survival time and their interaction. A separate GLM was constructed to examine if there was an interaction effect of CT and age group on survival.

To ensure that several tumor-related variables were not related to our variables of interest and confounding our observed patterns (age group and treatment modality), we constructed three GLMs. The first model was structured to examine the interaction of tumor stage with age group, CT, and radiation or surgery. The second and third models used the same form substituting tumor stage and lymph node involvement for tumor grade, respectively. The *p*-values of these three models were corrected for alpha inflation using the Benjamini–Hochberg procedure [22].

## 3. Results

**Figure 1:**
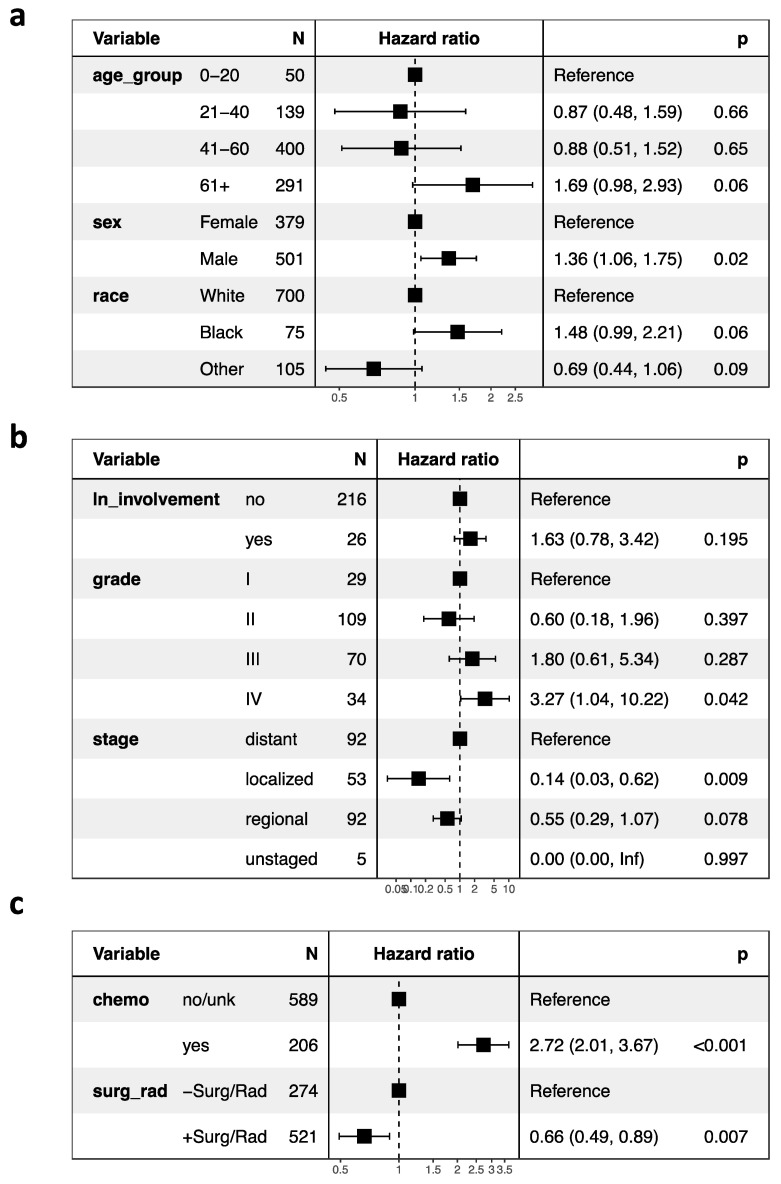
Cox proportional hazard ratios and forest plots of (**a**) patient demographics, (**b**) tumor characteristics, and (**c**) treatment regimen of the study population. Abbreviations: ln, lymph node; chemo, chemotherapy; surg, surgery; rad, radiation. Values in parentheses indicate the 95% confidence intervals.

We report survival interactions between age at diagnosis, race, sex, CT, radiation, surgery, tumor grade and lymph node involvement using Cox proportional hazard ratio analysis (Figure 1a–c). When compared to whites, there were no significant correlations between CSS and black (HR, 1.48 95% CI, 0.99–2.21; *p* = 0.06) or “other” races (HR, 0.69; 95% CI, 0.44–1.06; *p* = 0.09; Figure 1a). In addition, male patients had significantly decreased CSS compared to females (HR, 1.36; 95% CI, 1.06–1.75; *p* = 0.02). As previously reported, however, after case-matching sex for tumor grade and stage, these trends are no longer statistically significant (*p* > 0.05) [23].

**Figure 2:**
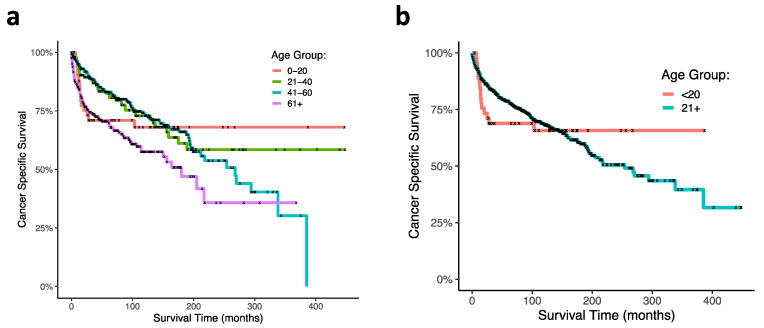
Kaplan–Meier curves of ONB patient cancer-specific survival within age at diagnoses. (**a**) Ages at diagnoses were stratified at 0–20 years, 21–40 years, 41–60 years, and greater than 60 years. (**b**) Survival curve comparing 0–20-year-old age group with patients greater then 20 years old at diagnosis.

Regarding patient age at diagnosis, overall, geriatric patients trended towards the lowest CSS among other age groups (HR, 1.69 95% CI, 0.98–2.93; *p* = 0.06). A significant effect of age at diagnosis on CSS was observed using Kaplan–Meier and log ranked analyses (*p* < 0.0001; Figure 2a). Of note, the pediatric age group (0–21 years) and the geriatric age group (>60 years) had the lowest CSS, notably within the first 100 months following diagnosis, while CSS of the middle-aged group (22–40 and 41–60 years) was greater at these same time points. Interestingly, pediatric mortality plateaued after around 25 months, and survival was the highest among all age groups thereafter. To more closely analyze the pediatric age group through time, we consequentially conducted a GLM discretized through time comparing the 0–20 age group against all other age groups and observed a significant age by time interaction: the 0–20 age group had the lowest CSS within the first 100 months but the highest CSS following this time point (*p* = 0.000126). Survival curves of this analysis are presented (Figure 2b).

**Figure 3:**
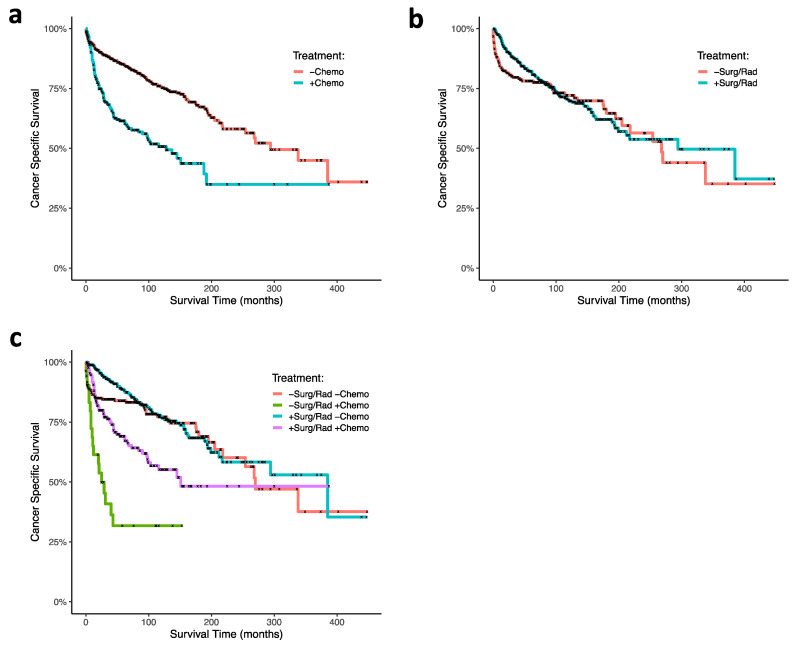
Kaplan–Meier curves of ONB patient cancer-specific survival associated (**a**) with and without chemotherapy treatment, (**b**) with and without radiation therapy plus and surgery, and (**c**) combinations of chemotherapy, radiation, and surgery. Abbreviations: chemo, chemotherapy; surg, surgery; rad, radiation.

**Figure 4:**
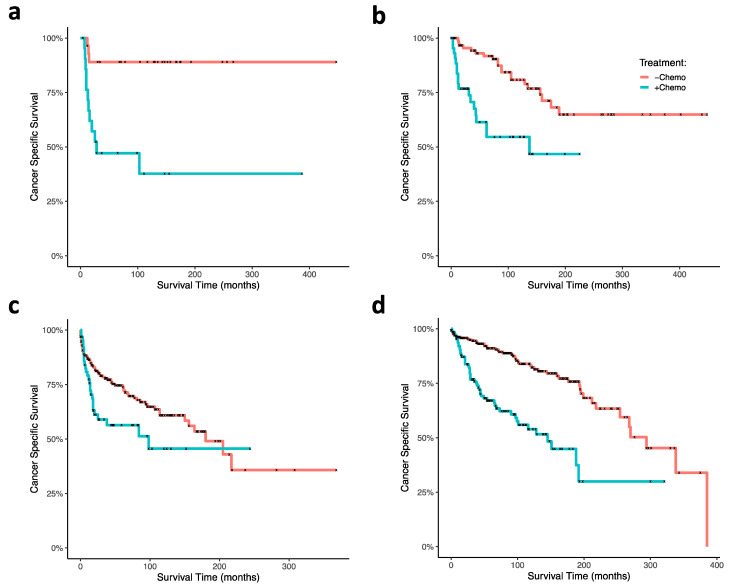
Kaplan–Meier curves of ONB patient cancer-specific survival within age at diagnoses and associated chemotherapy treatment status. Ages at diagnoses were stratified at (**a**) 0–20 years, (**b**) 21–40 years, (**c**) 41–60 years, (**d**) and greater than 60 years. Abbreviations: chemo, chemotherapy.

Using cox proportional hazard ratio analysis, RT and surgery were associated with increased CSS (HR 0.66; 95% CI, 0.49–0.89; *p* = 0.007), and chemotherapy was associated with decreased CSS (HR, 2.72; 95% CI, 2.01–3.67; *p* < 0.001; Figure 1c). Our Kaplan–Meier survival analyses support these findings: CT was associated with poor survival outcomes in ONB patients (*p* < 0.0001; Figure 3a), and RT and surgery together were associated with improved survival early timepoints (approximately with 100 months), although had no significant effects at the final time points (*p* = 0.3; Figure 3b). In analyzing combinations of patients that received CT, or RT and surgery, CT was again associated with poor survival outcome; patients receiving CT but neither RT nor surgery demonstrated the poorest CSS (*p* < 0.0001; Figure 3c). CT treatment was associated with decreased survival outcome within each age group (0–20 (*p* = 0.0004), 21–40 (*p* = 0.0002), 41–60 (*p* < 0.0001), 61+ (*p* = 0.02); Figure 4a–d). In our GLM analyses, however, we found no interaction effect of age group and CT through time (*p* = 0.0876). Although tumor grade and tumor stage were correlated with decreased CSS (Figure 1b), results from our GLMs show no interaction effects of tumor stage, tumor grade, and lymph node involvement, with either CT, radiation and surgery, or age group (Table 1).

## 4. Discussion

Previous studies have generated mixed results on survival outcomes of different ONB patient age groups, most notably in pediatric populations. In this SEER dataset analysis, we observe in pediatric and geriatric patients that the cancer-specific survival is significantly lower than other age groups approximately within the first 50 months. However, through time (over the course of 500 months) the pediatric patient survival curve plateaus and maintains the highest CSS of all groups at the endpoint. These findings suggest that comparisons of ONB patient survival data in age groups are not static and should be interpreted by analyzing dynamical trends over time.

Due to the low incidence of ONB, especially in pediatric patient populations, the effects of ONB on the survival of patient age groups remain speculative [6,17]. The incidence of ONB in children and adolescents less than 20 years of age is usually estimated to be less than 10% of all ONB cases [6]. The rarity of this disease in children presents a particular challenge in analyzing pediatric patients in more discrete age categories (e.g., <10 years versus <21 years). Consequentially, we analyzed one pediatric age group (ages 0–20 years; N = 50) to maintain statistical power. Interestingly, age has been shown to be a prognostic predictor of ONB survival outcomes, although studies have generated mixed results on whether children or adults are equally or differently affected by ONB. Previous studies have demonstrated that children and adolescents are more severely impacted by ONB [9,10,11,12,13]. Others have postulated that there is no significant difference between child and adult survival [6,14,15]. For example, Kadish et al. and Bisogno et al. have previously shown that ONB may be more aggressive in younger patients [9,13], while Yin et al. recently reported in historical analysis that ONB patients aged ≤20 years had 5-year CSS (72%) equal to patients aged >20 years (78%) [6]. Based on our results, we hypothesize that child (and perhaps geriatric) ONB patients may be most vulnerable to ONB or cancer-associated morbidities within the first 25–100 months following diagnosis, and that surviving this early time period may be a predictor of long-term survival in pediatric patients.

We demonstrate an association between CT and decreased CSS in all age groups, although there is no significant difference in this association between age groups. Regarding the observed association of patient mortality and CT treatment, it is possible that the sequence of CT treatment relative to surgery or RT may play a role in toxicity and survival outcome. For example, the toxicity of adjuvant or adjunct CT may be especially enhanced in patients who have already had surgery or been treated with RT. Neoadjuvant CT treatment strategies, on the other hand, could delay necessary surgery and RT treatment, resulting in increased mortality and morbidity. Moreover, perhaps in some cases, ONB does not respond very well to CT, and thus effects of drug toxicity and tumor morbidity are amplified. Given the rarity of the disease and that use of CT in the ONB standard of care is controversial, it is possible that CT sequence strategies and dosages have not been well established.

Many factors may be linked to the observed CSS association between age at diagnosis and CT treatment. Indeed, variables related to disease severity, such as tumor grade, stage, and lymph node involvement, could be correlated with CT treatment or age at diagnosis. Unlike many other studies, however, we accounted for these variables in mixed-effects analyses and report no significant interactions of disease severity on age at diagnosis or treatment modality. Certainly, other variables not analyzed could affect survival outcomes (e.g., genetic factors), and CT may have clinical benefits independent of patient survival not demonstrated here.

Of note, radiation and surgery were associated with improved survival in our Cox proportional hazard models, and survival analysis shows that through time, this treatment has a prominent effect within the first 100 months following diagnosis. However, when all available timepoints are taken into consideration (through 500 months), treatment effects of radiation and surgery are less prominent statistically. Thus, it is likely that treatment effects are most notable at earlier timepoints and may become less evident at later timepoint due to other factors not reported in SEER, such as recurrent disease or comorbidities.

It is important to note that despite the improved survival outcomes associated with RT and surgery, some cancer patients are not good candidates for RT or surgery and therefore only received CT. Considering our findings regarding CT and survival, understanding the therapeutic effects and long-term outcomes of CT in ONB patients is especially important.

Although it is difficult to account for all variables in retrospective studies, we analyzed available variables that have been shown to be prognostic predictors in ONB [5,6,16,21,24,25,26]. In addition, some variables are not available for every patient included in this study, which may pose a study limitation. However, analyzing such variables still provides valuable insight, and we reported N-values for each variable used in our analyses (Figure 1).

To account for variation and advancement of cancer therapies since the availability of the SEER dataset, we analyzed the most recent data for which CT status was available, which we believe represents the current state of oncologic treatment regimes, as cancer treatments, as well as skull base and craniofacial surgical techniques, have significantly changed since SEER began recording data. In addition, CT data provided by SEER only report CT treatment in two distinct categories: “yes” or “no/unknown.” Although there is a possibility of misclassification in the “no/unknown” category, our data show survival differences between “yes” and “no/unknown” patient groups.

## 5. Conclusions

In conclusion, our results demonstrate variations in CSS through time; ONB pediatric patients had the worse survival outcomes at early timepoints, and the best survival outcomes at later timepoints relative to other patient age demographics, indicating that such ONB survival statistics should be considered dynamic through time. CT treatment was associated with decreased CSS in all age groups through time, and radiation and surgery were associated with increased CSS within the first 100 months post-diagnosis. Moreover, these observations were independent of measurements associated with advanced disease. Further investigation of long-term outcomes associated with ONB patient age and treatment modality, especially CT, within and between age stratifications are needed to elucidate the underlying causes of our observations.

## Figures and Tables

**Table 1:** Generalized linear model statistics demonstrating CSS interactions between ONB disease severity (tumor stage, grade, and lymph node involvement) and chemotherapy, radiation and surgery or patient age at diagnosis.

Disease Severity	Chemotherapy	Radiation and Surgery	Patient Age
Tumor Grade	*p* = 0.83	*p* = 0.18	*p* = 0.415
Tumor Stage	*p* = 0.938	*p* = 0.415	*p* = 0.953
Lymph node metastasis	*p* = 0.649	*p* = 0.83	*p* = 0.953

## Data Availability

All data are made available in the manuscript. The original dataset is publicly available and may be accessed at https://seer.cancer.gov/ (accessed on 20 March 2020).
